# Flame Retardancy and Thermal Degradation Behaviors of Thiol-Ene Composites Containing a Novel Phosphorus and Silicon-Containing Flame Retardant

**DOI:** 10.3390/polym14040820

**Published:** 2022-02-20

**Authors:** Fangyi Wu, Xiaohui Bao, Jiangbo Wang

**Affiliations:** School of Materials and Chemical Engineering, Ningbo University of Technology, Ningbo 315211, China; w18758809619@163.com (F.W.); bxh19883979039@163.com (X.B.)

**Keywords:** flame retardancy, thiol-ene, thermal degradation, kinetics, activation energy

## Abstract

In this article, a novel phosphorus and silicon-containing flame retardant (DOPO-V-PA) was synthesized via condensation reaction and then added into thiol-ene (TE) to prepare a flame-retardant composite. The results of cone calorimeter measurement demonstrated that, compared with pure TE, 22.7% and 53.2% reduction of TE/DOPO-V-PA (thiol-ene/9,10-dihydro-9-oxa-10-phosphaphenanthrene-10-oxide-vinyltrimethoxysilane-phenyltrimethoxysilane-(3-aminopropyl)trimethoxysilane copolymer) was found for the peak heat release rate (PHRR) and total heat release (THR), respectively. The thermal degradation of TE composites was investigated by the TGA measurement under non-isothermal conditions, and kinetic parameters were both calculated by the Kissinger and Flynn-Wall-Ozawa methods. It was indicated that the activation energies of TE at conversions exceeding 50% were enhanced by the incorporation of DOPO-V-PA for the whole conversion range.

## 1. Introduction

UV polymerization is an effective method, which allows for processing under environmental conditions and provides temporal and spatial control of microstructure formation. It has advantages including low energy consumption, less environmental pollution, a low process cost, high chemical stability, and high efficiency [1,2,3]. UV photopolymerization is used in several industrial fields such as in inks, coatings, optical equipment, adhesives and dental restoration, and electrical and electronic systems. It can also use lithography technology to promote the preparation of complex three-dimensional structures [4,5,6,7,8].

The thiol-ene polymerization system, as a new photopolymerization system, follows a mechanism involving light induced step-by-step growth. It has the unique characteristics of curing without a photoinitiator, reducing oxygen inhibition and rapid curing and improving adhesion and low shrinkage [9,10]. In addition, one of the main characteristics of thiol-ene photopolymerization is that almost any type of ene monomer can participate in polymerization. Due to this, with its unique reaction mechanism and chemical structure, photopolymerized thiol-ene networks have attracted extensive attention in both academia and various industries [11,12,13,14,15]. However, the flammability of thiol-ene material greatly limits its application adaptability in many places. Therefore, thiol-ene polymers need to be flame retardant modified to broaden their potential industrial applications. 

Some approaches have been used to improve the flame retardancy of thiol-ene. Both allyldiphenyl phosphine oxide (ADPPO) and 4-vinylphenyl boronic acid are used as flame retardants in the reported methods of developing flame retardant thiol-ene [16,17]. Unfortunately, they all suffer from the disadvantages of low flame retardant efficiency and the release of toxic and corrosive smoke during combustion.

Therefore, it is urgent to develop a new thiol-ene flame retardant system with high flame retardant efficiency, high thermal stability, and environmentally friendly properties.

In our previous research, silicone flame retardant was added to thiol-ene polymer and achieved good flame retardant effect, while DOPO (9,10-Dihydro-9-oxa-10-phosphaphenanthrene-10-oxide), another common organophosphorus flame retardant, was also demonstrated to effectively enhance the flame retardancy of the materials [18,19]. Meanwhile, phosphorus and silicon showed good synergistic effect on fire resistance for epoxy resins, PET (polyethylene terephthalate), etc. [20,21,22,23,24]. When phosphorus and silicon are on the same molecular structure, the flame retardant polymer has the best effect [25,26]. In this article, a new phosphorus and silicon-containing flame retardant DOPO-V-PA (DOPO-vinyltrimethoxysilane-phenyltrimethoxysilane-(3-aminopropyl)trimethoxysilane copolymer) was firstly synthesized, and the role of the DOPO-V-PA flame retardant in the combustion behavior and thermal degradation of thiol-ene composites was investigated in detail. Moreover, two kinds of thermal degradation kinetic methods were used to study the kinetic parameters (especially activation energy) of the composites.

## 2. Materials and Methods

### 2.1. Materials

2,2′-Azobisisobutyronitrile (AIBN), (3-aminopropyl)trimethoxysilane (APS) and tetramethylammonium hydroxide (TMAOH) were supplied by Alfa Aesar Chemical Reagent Co. Ltd. (Tewksbury, MA, USA). Vinyltrimethoxysilane (VTMS), benzene (reagent grade), ethyl alcohol (EtOH, 95%), pentaerythritol allyl ether (TAE) and 2,2-dimethoxy-2-phenylacetophenone (DMPA) were supplied by Sigma-Aldrich Reagent Co. Ltd. (St. Louis, MO, USA). 9,10-Dihydro-9-oxa-10-phosphaphenanthrene-10-oxide (DOPO) was supplied by TCI Development Co., Ltd. (Tokyo, Japan). Phenyltrimethoxysilane (PTMS) was supplied by Gelest Chemical Reagent Co., Ltd. (Morrisville, PA, USA). Trimethylolpropane tris(3-mercaptopropionate) (3T) was supplied by Bruno Bock Chemische Fabrik Gmblt & Co. (Marschacht, Germany). None of the chemicals underwent any further purification.

### 2.2. Preparation of DOPO-V-PA

14.8 g VTMS, 21.6 g DOPO and 100 mL benzene were added into a three port flask with dropping funnel, condenser and mechanical stirrer. Under the protection of nitrogen, the slurry was mixed well by mechanical stirrer, and then the temperature was slowly raised to 80 °C. 0.1 g AIBN was dissolved in 50 mL benzene to form a solution, and it was slowly dropped into the above reactor within 2 h. The reaction temperature was maintained for 24 h. After that, the product was purified by filtration, and then the solvent benzene was removed by rotary evaporator to obtain a colorless liquid product 9,10-dihydro-9-oxa-10-phosphaphenanthrene-10-oxide-vinyltrimethoxysilane copolymer (DOPO-V).

**Figure 1:**
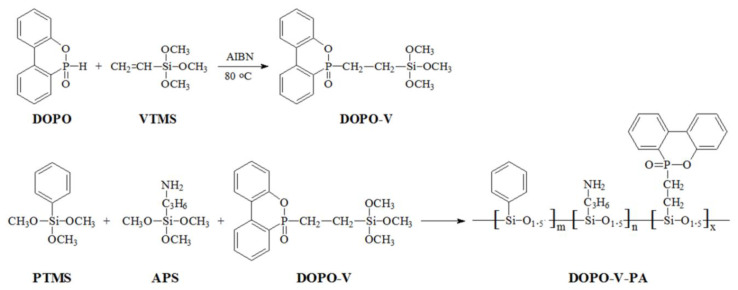
Preparation route of DOPO-V-PA.

The preparation of DOPO-V-PA consists mainly of hydrolysis and condensation reactions; its reaction equation is shown in Figure 1. The specific preparation process includes adding 75 mL ethanol, 25 mL distilled water and 1 mL TMAOH into a 250 mL flask and stirring and mixing evenly. Then, a mixture of PTMS, DOPO-V and APS with a molar ratio of 70%:20%:10% was added, and the mass of the mixture was 10% of the total weight of the solution. After 8 h, stirring was stopped and the mixture was left at room temperature overnight. The supernatant was poured out, washed and filtered with distilled water/ethanol mixture (volume ratio 1:3) and ethanol respectively, and the solid sample was dried in a vacuum at room temperature for 20 h to obtain 9,10-dihydro-9-oxa-10-phosphaphenanthrene-10-oxide-vinyltrimethoxysilane-phenyltrimethoxysilane-(3-aminopropyl)trimethoxysilane copolymer (DOPO-V-PA) product [27].

### 2.3. Preparation of Thiol-Ene Composites

**Figure 2:**
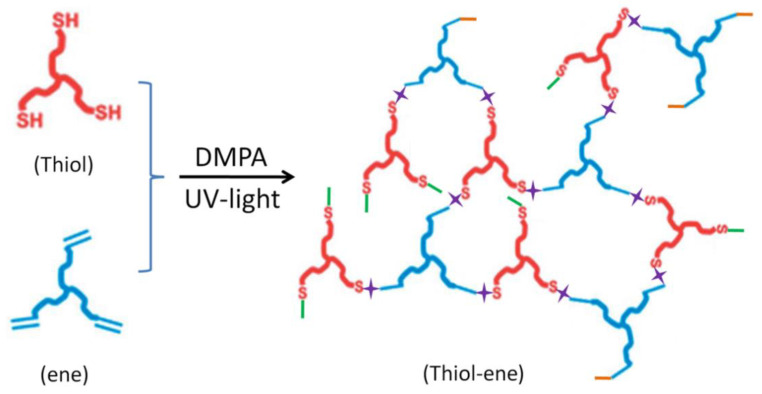
Chemical structure of thiol-ene.

The preparation process of TE/DOPO-V-PA (flame retardant TE, FRTE) composites is as follows. The photoinitiator DMPA was dissolved in 3T and mixed for 30 min. Then, TAE was added and stirred evenly. Bubbles were removed by ultrasonic oscillation for 30 min and the sample was poured into a glass container for sample preparation. The film was cured under the fusion UV curing line system of D bulb (400 W/cm^2^, belt speed 3 m/min, irradiance 3.1 W/cm^2^). During the experiment, the equivalence ratio of thiol to ene compounds was 1:1, and DOPO-V-PA and DMPA accounted for 5 wt% and 1 wt% of the total mass, respectively. The chemical structure of thiol-ene was shown in Figure 2. Meanwhile, pure TE samples were prepared under the same process conditions for comparison.

### 2.4. Characterization and Measurement

Cone calorimeter measurements were in accordance with ASTM E1354 and performed on an FTT cone calorimeter (Fire Testing Technology Ltd., East Grinstead, West Sussex, UK). The specimen size is 100 mm × 100 mm × 3 mm and the heat flow is 50 Kw/m^2^. All specimens were tested three times. Flame retardancy of the tested specimens was also examined in accordance with UL-94 method on a CZF-3 horizontal and vertical flammability tester (Nanjing Jiangning Analytical Instrument Co., Ltd., Nanjing, China). A thermogravimetric analysis (TGA) test was performed on TA instrument Q5000 thermogravimetric analyzer (TA instrument company, New Castle, DE, USA). We weighed about 10 mg of the sample, placed it in nitrogen atmosphere, and raised the temperature from 50 °C to 600 °C at a heating rate of 10 °C/min.

## 3. Results and Discussion

### 3.1. Flame Retardancy

**Figure 3:**
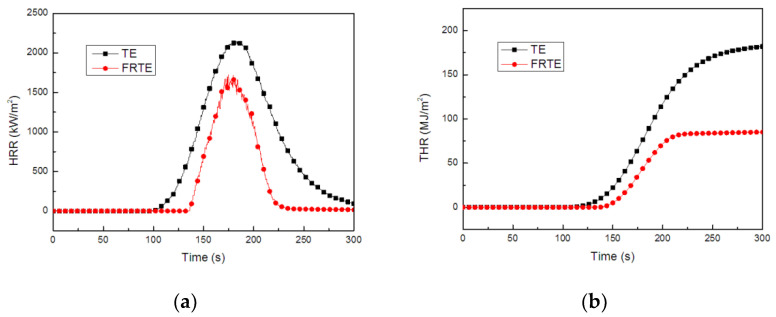
The heat release rate (**a**) and total heat release (**b**) curves for TE composites.

**Figure 4:**
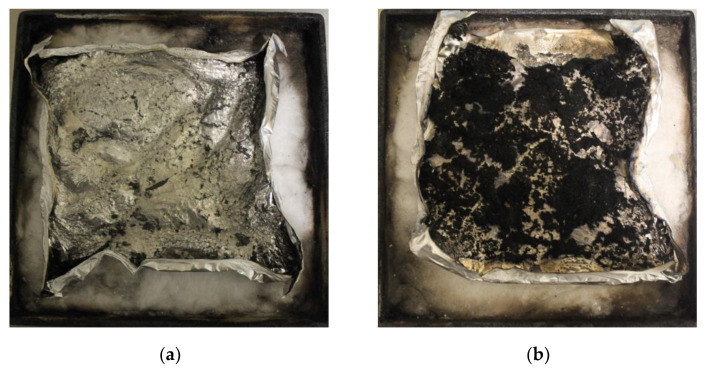
Residual char images of TE (**a**) and FRTE (**b**) after CONE measurement.

Figure 3 showed the heat release rate (HRR) and total heat release (THR) curves versus time for TE composites. For FRTE, a 22.7% and 53.2% reduction was found for peak heat release rate (PHRR) and THR, respectively, compared with the pure TE system. Meanwhile, it is worth noting that the ignition time of FRTE is longer than that of pure TE. The UL-94 grade of pure TE is V-2 grade, and the flame retardant grade of FRTE is increased to V-0 grade. This is mainly due to the addition of DOPO-V-PA, which changes the combustion process of polymer. As shown in Figure 4, with the addition of DOPO-V-PA, the amount of residue char after combustion of the thiol-ene polymer increased significantly. Since the char layer has a three-dimensional network structure, it is very dense and difficult to burn.

### 3.2. Thermal Stability

**Figure 5:**
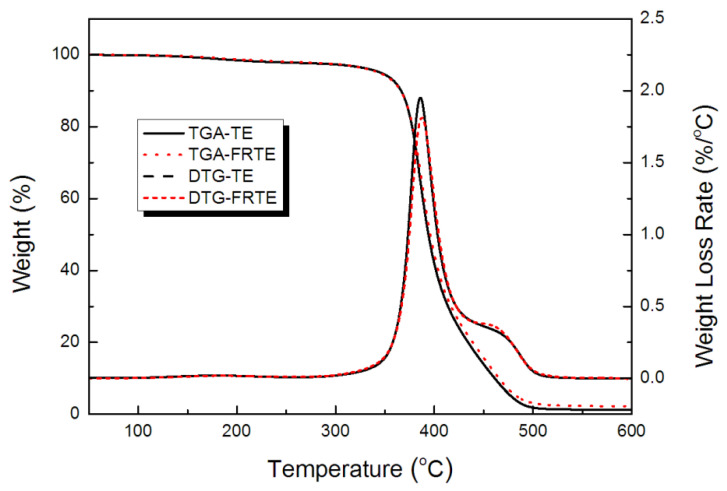
Thermal stability of TE composites.

To study the effect of DOPO-V-PA on TE thermal degradation, the TGA and DTG curves of TE composites at 10 °C/min under nitrogen atmosphere were plotted in Figure 5, and a detailed data summary has been listed in Table 1. The onset degradation temperature (*T_5wt%_*) of the FRTE composite was lower than that of pure TE. This was caused by the degradation of DOPO-V-PA, which was good for protecting the polymer matrix from heat flux. *T_max_* of the composites containing DOPO-V-PA was slightly higher than that of pure TE. Surprisingly, the char yield of the FRTE was increased to 2.66 wt% from 1.33 wt% of pure TE. It might be caused by the two reasons. One of them was that the char layer from DOPO-V-PA prevented the release of the degradation products of TE. The other was that DOPO-V-PA could promote the formation of the compact char layer of TE.

### 3.3. Thermal Degradation Kinetics

**Figure 6:**
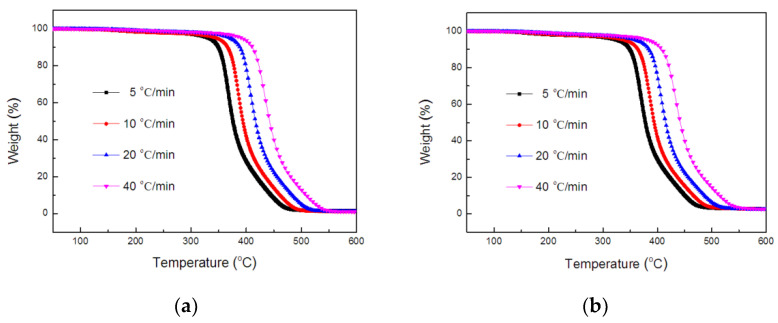
TGA curves of TE (**a**) and FRTE (**b**) composites.

**Figure 7:**
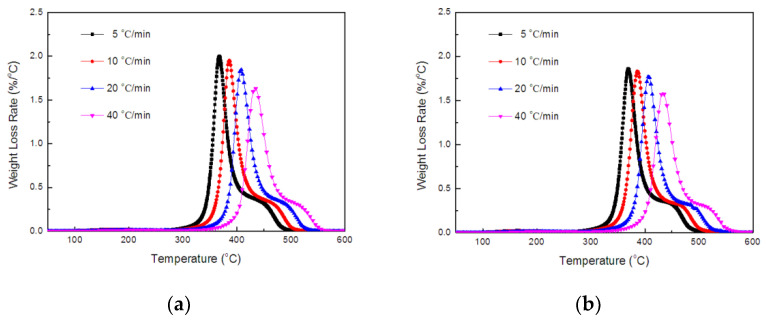
DTG curves of TE (**a**) and FRTE (**b**) composites.

Thermogravimetric analysis was performed at heating rates of 5, 10, 20, and 40 °C/min under a nitrogen gas flow. Figure 6 and Figure 7 show the TGA and DTG curves of TE composites. The thermal degradation process was studied by a kinetic parameters measured with TGA experimental data.

The thermal degradation process is generally described as a generalized chemical bonds breaking process composed of primary and secondary degradation events. However, there are many influencing factors, including material composition, temperature changes, and possible simultaneous chemical reactions, which lead to very complex and difficult to analyze alone [28,29].

Thermal degradation kinetic analysis is one of the potential solutions for this problem. The kinetic parameters for the whole degradation process were calculated by the Kissinger method. Its kinetic equation can be expressed as [30]:

(1) $$\ln(\frac{\beta }{T_{\max}^{2}})=\ln(\frac{AR}{E})-\frac{E}{RT_{\max}}$$

where *β* is the heating rate, *T_max_* is the peak rate temperature, *A* is the pre-exponential factor, *R* is the gas constant, and *E* is the activation energy.

**Figure 8:**
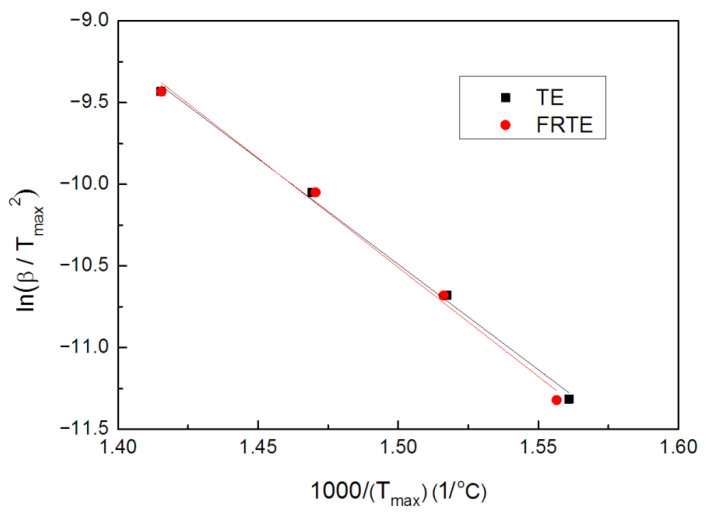
$\ln(\frac{\beta }{T_{\max}^{2}})$ vs. $\frac{1}{T_{\max}}$ curves of TE and FRTE.

Figure 8 is the fitting curve of $\ln(\beta /T_{\max}^{2})$ against 1/$T_{\max}$. It can be seen that the fitting line has a high linear correlation coefficient. This demonstrates that the Kissinger method is very suitable for this research system.

According to Equation (1), the activation energies of TE composites were calculated. The resulting data are listed in Table 2. First of all, it is worth mentioning that the fitting degrees (R2 values) of TE and FRTE fitting lines were 99.57% and 99.08% respectively. The activation energy of thermal degradation of pure TE was 107.4 kJ/mol and the value of FRTE increased to 111.1 kJ/mol, revealing that the DOPO-V-PA enhanced the thermal stability of TE matrix. An especially small amount of DOPO-V-PA increased the activation energy by about 3.7 kJ/mol, indicating that the FRTE with higher char yield had a higher activation energy of thermal degradation.

Adding a small amount of DOPO-V-PA increases the activation energy by about 3.7 kJ/mol. These results indicate that FRTE has higher activation energy of thermal degradation by virtue of high char residue rate.

The Flynn–Wall–Ozawa method is not only a typical model free method but is also a relatively simple and convenient method. It only needs the data of weight loss versus temperature of TGA curve at different heating rates in order to quickly calculate and determine the activation energy of the system.

The dynamic equation of Flynn–Wall–Ozawa method is as follows [31,32]:

(2) $$\mathrm{lg}(\beta )=\mathrm{lg}AE/g(a)R-2.315-0.457\frac{E}{RT}$$

**Figure 9:**
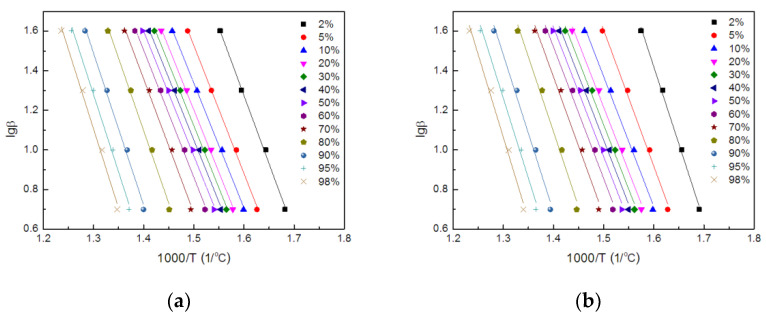
The plots of lg(β) vs. 1000/*T* of TE (**a**) and FRTE (**b**).

The plots of lg(β) versus 1000/*T* were shown in Figure 9, suggested that the fitting straight lines were nearly parallel and thereby indicating the applicability of this method to TE composites within the conversion range studied.

**Figure 10:**
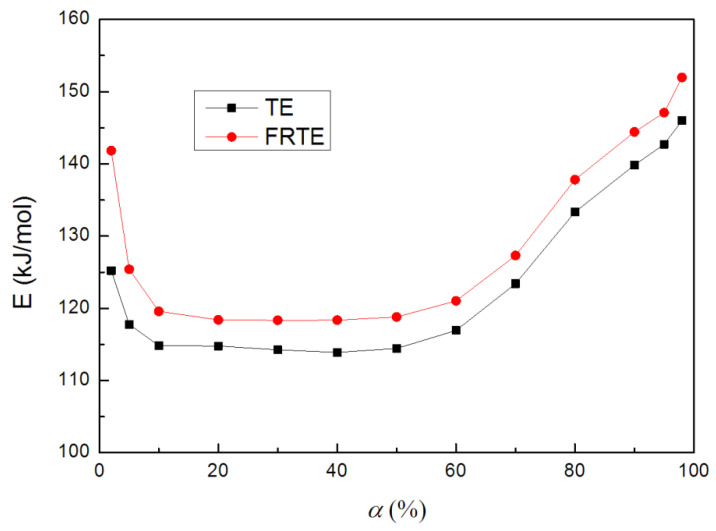
Activation energy curves of TE and FRTE by the Flynn-Wall-Ozawa method.

For given conversion values, activation energy can be calculated from the plots in various extent of conversion according to the Equation (2). For this study, conversion values from 2% to 98% have been used. Activation energies, corresponding to the different conversions in nitrogen atmosphere, are shown in Figure 10.

It could be found that the activation energies of TE and FRTE were both decreased with the increase of conversion at low conversions, whereas the higher activation energies could be observed at high conversions. Moreover, these values showed that the presence of DOPO-V-PA enhanced the activation energies of TE in whole degradation, which was related to the results obtained from the Kissinger method. The average activation energy of pure TE was 124.4 kJ/mol, which was lower than that of FRTE (130.3 kJ/mol). The higher activation energy of FRTE was due to the formation of better thermal stability of the char layer.

## 4. Conclusions

In summary, an incorporation of a small amount of DOPO-V-PA (5 wt%) reduced the PHRR (22.7%) and THR (53.2%) of TE composite, and it was demonstrated that DOPO-V-PA was an effective flame retardant for TE. The results of our measurements showed that DOPO-V-PA improved the char residual amounts of TE. Furthermore, the Kissinger and Flynn-Wall-Ozawa methods were both applied to study the thermal degradation kinetics of TE composites. Compared with pure TE, the activation energies of TE at conversions exceeding 50% *E* grow, but at lower conversions activation energy reduces by the incorporation of DOPO-V-PA due to the formation of better thermal stability of the char layer.

## Figures and Tables

**Table 1:** TGA data of TE composites.

Sample	Temperature (°C) ^1^		Peak Rate (wt%/°C) ^2^	Residue Char (wt%) ^3^
	*T_5wt%_*	*T_max_*		
TE	345.7	385.9	1.95	1.33
FRTE	341.0	386.4	1.83	2.66

^1^ Measurement error is 0.1 °C; ^2^ Measurement error is 0.01 wt%/°C; ^3^ Measurement error is 0.01 wt%.

**Table 2:** Kinetic data for TE and FRTE degradation by the Kissinger method.

	Temperature (°C)				*E* (kJ/mol)	ln*A* (1/min)
	5 °C/min	10 °C/min	20 °C/min	40 °C/min		
TE	367.5	385.9	407.5	433.4	107.4	11.4
FRTE	369.3	386.4	406.9	433.3	111.1	12.1

## Data Availability

The data used to support the findings of this study are available from the corresponding author upon request.
